# Cross-country evidence on the association between contact tracing and COVID-19 case fatality rates

**DOI:** 10.1038/s41598-020-78760-x

**Published:** 2021-01-25

**Authors:** Abdullah Yalaman, Gokce Basbug, Ceyhun Elgin, Alison P. Galvani

**Affiliations:** 1 Department of Business Administration, Eskisehir Osmangazi University, Meselik Campus, 26040 Eskisehir, Turkey; 2 SKK Graduate School of Business, Sungkyunkwan University, 25-2, Sungkyunkwan-ro, Jongro-gu, Seoul, 03063 South Korea; 3 Department of Economics, Bogazici University, Natuk Birkan Building, 34342 Bebek, Istanbul, Turkey; 4 Center for Infectious Disease Modeling and Analysis, Yale School of Public Health, Yale University, New Haven, CT 06520 USA

**Keywords:** Health policy, Public health, Epidemiology

## Abstract

The coronavirus disease (COVID-19) outbreak has killed over a million people since its emergence in late 2019. However, there has been substantial variability in the policies and intensity of diagnostic efforts between countries. In this paper, we quantitatively evaluate the association between national contact tracing policies and case fatality rates of COVID-19 in 138 countries. Our regression analyses indicate that countries that implement comprehensive contact tracing have significantly lower case fatality rates. This association of contact tracing policy and case fatality rates is robust in our longitudinal regression models, even after controlling for the number of tests conducted and non-pharmaceutical control measures adopted by governments. Our results suggest that comprehensive contact tracing is instrumental not only to curtailing transmission but also to reducing case fatality rates. Contact tracing achieves the early detection and isolation of secondary cases which are particularly important given that the peak in infectiousness occurs during the presymptomatic phase. The early detection achieved by contact tracing accelerates the rate at which infected individuals receive medical care they need to maximize their chance of recovery. In addition, the combination of reduced transmission and more rapid recovery diminishes the burden on the healthcare system which in turn ensures that the resources remain available for individuals who do become infected.

## Introduction

The ongoing coronavirus disease (COVID-19) outbreak has spread to 213 countries and territories, causing more than 47,000,000 cases and over 1.2 million deaths as of November 2020. A considerable fraction of patients shows severe pneumonia-like symptoms who require critical care^1^. Compounding the severity of the disease are the challenges that have hampered its control. Specifically, silent transmission from a combination of the presymptomatic phase and from cases that remain asymptomatic^2^ account for the majority of COVID-19 spread^3^. Given that viral shedding peaks during the presymptomatic phase^4^, control measures that rely on symptom detection are inadequate^3^^.^ Therefore, testing and contact tracing are both crucial to detect and isolate positive cases to mitigate the outbreak and to diminish the burden on the healthcare system^5^.

COVID-19-related death rates vary across countries^6^. Anecdotal evidence suggests that countries that have controlled the epidemic and that have relatively lower death rates have conducted ample testing and comprehensive contact tracing (e.g., South Korea, Germany)^7–9^. Although the importance of contact tracing has been discussed extensively in the popular press and examined in modeling studies, its effectiveness in limiting fatalities has not yet been evaluated statistically with country-level data^10–12^.

Contact tracing involves identifying, testing and quarantining the contacts of an index case. It had been used as an effective policy in the control of infectious diseases, including severe acute respiratory syndrome^13^. In response to COVID-19, countries vary regarding the extent of their contact tracing policy. While some countries have implemented limited contact tracing throughout the pandemic (e.g., Estonia), others have been conducting comprehensive contact tracing by identifying and isolating all contacts since detection of its first case (e.g., Slovenia). Other countries have revised their policies over time. For instance, Denmark did not implement contract tracing during the first two months of the outbreak, implemented contact tracing for some of the cases during the following six weeks (from March up to the third week of April), and finally adopted contact tracing for all cases since the third week of April.

The role of contact tracing in reducing the transmission of COVID-19 has been demonstrated in Taiwan and China^14,15^. In addition, modeling studies have examined the efficacy of contact tracing interventions^16,17^. However, data-driven cross-country examination is lacking. In this paper, using data on diagnostic tests, non-pharmaceutical measures, healthcare capabilities, population characteristics, and economic indicators from 138 countries, we quantitatively evaluate the association between different contact tracing policies and COVID-19 case fatality rates.

## Methods

### Study variables and data sources

In our analysis, we considered the number of total tests (per million), stringency score for non-pharmaceutical measures, the number of physicians and hospital beds, (both per 1000), log population and percentage of population with age over 70, percentage of smokers and diabetes (% of population aged 20 to 79), GDP per capita, and the magnitude of fiscal stimulus introduced over the pandemic. Our data come from publicly available sources. Appendix A provides detailed information on our study variables, as well as the data sources. Moreover, the descriptive statistics of the variables, including the mean, standard deviation, and minimum and maximum values are provided in Table 1. Data for our primary independent variable, contact tracing policy, were taken from the Oxford Covid-19 Government Response Tracker^18^. The Oxford Tracker provides a systematic cross-country, cross-temporal data collected and updated in real time that monitor how government responses to COVID-19 have changed throughout the pandemic. It keeps track of governments’ policies and public health measures across three main categories (i.e., containment and closure, economic response, and health systems) and creates a composite score for each category. In our analysis, we used countries’ daily contact tracing policy, coded using a 3-point Likert scale (0 = no contact tracing, 1 = limited contact tracing, not done for all cases, 2 = comprehensive contact tracing; done for all identified cases).

**Table 1:** Descriptive statistics.

Variables	N	Mean	SD	Min	Max
Age over 70	1636	5.864	4.207	0.526	18.49
Diabetes (% of population aged 20 to 79)	1739	8.662	4.470	0.990	23.36
# of hospital beds (per 1000)	1512	3.049	2.253	0.100	13.80
Percentage of smokers	1246	22.51	9.749	4	45.95
# of physicians (per1000)	1716	2.094	1.767	0.0140	8.422
Fiscal stimulus	1468	5.820	5.985	− 5	42.20
GDP per capita	1625	20.20	18.49	0.661	116.9
Case fatality rate	1528	0.092	0.236	0	0.9436
Case mortality rate	1841	8.107	24.47	0	589.3
Total tests (per million)	1630	5.138	14.73	0	162.4
Contact tracing	1378	1.491	0.663	0	2
Stringency Score	1395	56.80	24.13	0	100
Log-population	1840	15.23	2.494	6.696	21.09

The measure for the intensity of non-pharmaceutical controls adopted by governments was also taken from the Covid-19 Government Response Tracker. The Tracker keeps track of the containment policies (i.e., school closing, workplace closing, stay at home requirements) implemented by governments and provides a total stringency score (a higher score indicates more stringent measures).

We compiled COVID-19-related data on the number of tests, cases, and deaths as well as country-specific characteristics (population, the percentage of the population over 70 years old, GDP per capita, the percentage of smokers as well as people with diabetes in the overall population) from ourworldindata.org^19^. Data on countries’ pre-pandemic healthcare capabilities were taken from the World Development Indicators^20^ and national websites. Data on the fiscal stimulus packages introduced by governments during the pandemic to revive economies were taken from the COVID-19 Economic Stimulus Index^21^.

COVID-19-related data (the number of tests, cases, deaths, and contact tracing policy) for each country were taken from the day the first case was recorded in a country to October 3^rd^ for all countries. All data used in this study are aggregated in one dataset and are publicly available.

### Regression analysis

We ran panel estimations with the presence of time fixed effects to investigate the relationship between contact tracing policy and death rates^22,23^. We operationalized the case fatality rate as the number of deaths in closed cases (closed cases refer to those who have recovered or died). We used closed cases instead of total cases because taking the total case as the denominator may result in an underestimation of the death rate^24^.

For the longitudinal data analysis, we ran time fixed-effect regression models. The longitudinal data estimation is particularly useful in analyzing the dynamic lagged effects of different contact tracing policies on case fatality rates. By introducing time fixed-effects, we control for the effect of time. In addition, we include various country-level variables as controls. Moreover, for the longitudinal analysis, we transformed daily time-variant variables into biweekly observations. Specifically, we use the two-week period as the relevant frequency in our model and then regress the case fatality rate on lagged values of total test numbers, average stringency score, and average contact tracing score, where the lag refers to values in the previous two-week period. We used the 2-weeks interval in our analysis because previous research reported that the median number of days from the first symptom to death is 14 days^25^. In regression models, we use the fixed effects estimator, as dictated by the F and Hausman tests. Limited by the availability of the data, our cross-country longitudinal data span 138 countries in total.

## Results

In our study, the primary dependent variable is case fatality rates of COVID-19, operationalized as the number of deaths in closed cases. In our longitudinal data analysis, we regressed case fatality rates on four sets of predictors: testing policy variables (the number of tests and contact tracing policy), healthcare system capabilities (the number of hospital beds and physicians), country characteristics (population, the percentage of the population over 70 years old, GDP per capita, the percentage of smokers and people with diabetes in the population), economic measures against COVID-19 (fiscal stimulus) and stringency score (non-pharmaceutical public health control measures adopted by countries). Controlling for these variables, we examined the association between contact tracing policy in a 2-weeks period (T1) on case fatality rates in the following two weeks (T2).

Tables 2 presents the results from longitudinal data with time fixed effect regression models. The first model presents regression results for all countries including outlier observations for case fatality rates. Next, the second model presents results excluding outlier observations. We defined outliers as observations falling in the higher 5% fractions of the data for case fatality. The first model shows that contact tracing policy is marginally significantly and negatively associated with case fatality rates (β = −0.16, p < 0.10). The second model shows that contact tracing policy is significantly and negatively associated with case fatality rates (β = −0.13, p < 0.05).

**Table 2:** Longitudinal data regression models for case fatality rates on time 2 [T2].

Model	(1)	(2)
Variables	CFR	CFR
# of Biweekly Tests [T1]	− 0.0180***	− 0.0058*
	(0.0051)	(0.0033)
Stringency Score [T1]	0.0012	0.0013
	(0.0036)	(0.0019)
Contact tracing [T1]	− 0.1577*	− 0.1304**
	(0.0968)	(0.0678)
# of hospital beds (per 1000)	− 0.4193***	0.0212
	(0.0416)	(0.0187)
# of physicians (per1000)	− 0.1671***	− 0.1655***
	(0.0632)	(0.0343)
Age over 70	0.1459***	0.0993***
	(0.0257)	(0.0162)
Percentage of smokers	0.0526***	0.0023
	(0.0110)	(0.0049)
Diabetes (% of population aged 20–79)	− 0.1388***	− 0.0075
	(0.0307)	(0.0141)
Log population	0.1258**	0.1039***
	(0.0622)	(0.0241)
GDP per capita	0.0335***	− 0.0021
	(0.0046)	(0.0032)
Fiscal stimulus	0.0289*	− 0.0259***
	(0.0169)	(0.0085)
Constant	2.3763*	− 4.1035***
	(1.2824)	(0.5541)
Observations	731	634
Number of Countries	138	138

Robust standard errors in parentheses.

We estimate Model 1 including outliers. Model 2 excludes outliers.

***p < 0.01, **p < 0.05, *p < 0.10.

Our regression analysis with longitudinal data also shows that the number of physicians is significantly associated with case fatality rates, indicating that countries with a higher number of physicians have lower rates of case fatalities. In addition, countries implementing higher number of tests have lower rates of case fatalities. Moreover, countries with higher proportion of people over 70 years old have higher case fatality rates. Log population and the size of fiscal stimulus are also significantly associated with case fatality rates.

To improve the robustness of our findings from the longitudinal data analysis, we ran the same regression model using the mortality rate (the number of deaths per million) as a dependent variable. Model 3 and 4 in Table 3 presents coefficients including and excluding outliers, respectively. Overall the results show that the contact tracing policy is significantly and negatively associated with the mortality rate, controlling for a host of variables. In sum, the results from longitudinal data analyses suggest that countries that have utilized more comprehensive contact tracing have lower deaths from COVID-19, controlling for the number of tests conducted, country-specific characteristics, and other public health measures.

**Table 3:** Longitudinal data regression models for mortality rates on time 2 [T2].

Model	(3)	(4)
Variables	MR	MR
# of Biweekly Tests [T1]	− 0.0027	0.0021
	(0.0031)	(0.0030)
Stringency Score [T1]	0.0172***	0.0172***
	(0.0032)	(0.0033)
Contact tracing [T1]	− 0.4749***	− 0.2962***
	(0.0968)	(0.0929)
# of hospital beds (per 1000)	0.0891**	0.1473***
	(0.0408)	(0.0341)
# of physicians (per1000)	0.1213*	0.0246
	(0.0691)	(0.0527)
Age over 70	0.0916***	0.0568***
	(0.0270)	(0.0215)
Percentage of smokers	− 0.0375***	− 0.0171**
	(0.0085)	(0.0070)
Diabetes (% of population aged 20 to 79)	0.0770***	0.1019***
	(0.0180)	(0.0149)
Log-population	− 0.0042	− 0.0741**
	(0.0321)	(0.0297)
GDP per capita	0.0034	− 0.0040
	(0.0049)	(0.0037)
Fiscal stimulus	0.0015	− 0.0060
	(0.0127)	(0.0114)
Constant	− 2.0911***	− 1.3857**
	(0.7008)	(0.6682)
Observations	745	701
Countries	138	138

Robust standard errors in parentheses.

We estimate Model 3 including outliers. Model 4 excludes outliers.

***p < 0.01, **p < 0.05, *p < 0.10.

## Discussion

The primary goal of this study was to explore the effect of different contact tracing policy choices adopted by countries on decreasing COVID-19-related deaths. Our analyses with 138 countries showed that comprehensive contact tracing is a significant factor that is associated with reduced deaths from COVID-19. We provide empirical evidence that countries which utilized contact tracing more intensely have lower rates of case fatalities, controlling for pre-pandemic health capabilities, non-pharmaceutical public health controls, economic measures, and country-specific characteristics. Thus, our evidence with real-time country-level data confirms the anecdotal evidence on the effectiveness of contact tracing in limiting the human toll of the epidemic.

Our study has important policy implications. Effective national health systems and adequate government spending for public health are necessary to improve healthcare quality and decrease mortality rates under normal conditions^26^. However, in the case of a nationwide epidemic, additional interventions are needed to curtail the transmission of the virus and to diminish fatalities. Specifically, in the case of COVID-19 with high contagiousness, rapid and targeted responses are crucial. Thus, laboratory infrastructure for developing and producing diagnostic tests, flexible regulatory arrangements that allow rapid approval, strong decentralized systems to conduct and process tests, and widespread employment of epidemiologists to identify secondary cases are essential factors to consider for effective pandemic management. Early detection of index cases and identification and isolation of secondary cases through contact tracing are key to suppressing the epidemic.

Several limitations of our study need to be mentioned. First, our paper contributed to the understanding of the predictors of deaths from COVID-19; however, the pandemic still persists. Therefore, future research is very much needed to see the full picture of the predictors of case fatality rates when the pandemic ends. Second, due to data availability and reliability, we focused only on 138 countries in our analyses. Third, our analyses do not take the economic costs of different policies into account since country-level data on the costs of contact tracing are not available. Finally, the timing of the identification of secondary cases is vital for an effective contact tracing policy; however, we were not able to examine cross-country variation in timing due to data unavailability.

In this study, we investigated the association between contact tracing policies of countries and their case fatality rates. Our results suggest that comprehensive contract tracing is an effective policy, along with mass testing, for diminishing the burden on the healthcare system and speeding the rate at which infected individuals receive the medical care they need to maximize their chance of recovery.

## Supplementary information

Supplementary Information 1.

## References

[1] Moghadas SM (2020). Projecting hospital utilization during the COVID-19 outbreaks in the United States. Proc. Natl. Acad. Sci..

[2] Bai Y (2020). Presumed asymptomatic carrier transmission of COVID-19. JAMA.

[3] Moghadas SM (2020). The implications of silent transmission for the control of covid-19 outbreaks. Proc. Natl. Acad. Sci..

[4] He, Xi. *et al.* Temporal dynamics in viral shedding and transmissibility of COVID-19. *Nature Medicine* 26, 672–675, (2020). 10.1038/s41591-020-0869-5 32296168

[5] Salathé M (2020). COVID-19 epidemic in Switzerland: on the importance of testing, contact tracing and isolation. Swiss Medical Weekly.

[6] Liang LL (2020). Covid-19 mortality is negatively associated with test number and government effectiveness. Sci. Rep..

[7] Sun K, Viboud C (2020). Impact of contact tracing on SARS-CoV-2 transmission. Lancet Infect Dis.

[8] Normile D. Coronavirus cases have dropped sharply in South Korea. What’s the secret to its success? *Science*, March 17, 2020. https://www.sciencemag.org/news/2020/03/coronavirus-cases-have-dropped-sharplysouth-korea-whats-secret-its-success#. Accessed June 15, 2020

[9] Reintjes R (2020). Lessons in contact tracing from Germany. BMJ.

[10] Ferretti L (2020). Quantifying SARS-CoV-2 transmission suggests epidemic control with digital contact tracing. Science.

[11] Hellewell J (2020). Feasibility of controlling COVID-19 outbreaks by isolation of cases and contacts. The Lancet Global Health.

[12] MacIntyre CR (2020). Case isolation, contact tracing, and physical distancing are pillars of COVID-19 pandemic control, not optional choices. Lancet Infect Dis.

[13] Eames KTD, Keeling MJ (2003). Contact tracing and disease control. Proc. Biol. Sci..

[14] Cheng HY (2020). Contact tracing assessment of COVID-19 transmission dynamics in Taiwan and risk at different exposure periods before and after symptom onset. JAMA Intern Med.

[15] Bi, Q. *et al.* Epidemiology and transmission of COVID-19 in 391 cases and 1286 of their close contacts in Shenzhen, China: a retrospective cohort study. *Lancet Infect. Dis.* https://doi.org/10.1016/S1473-3099(20)30287-5 (2020) 10.1016/S1473-3099(20)30287-5 PMC7185944 32353347

[16] Kretzschmar ME (2020). Impact of delays on effectiveness of contact tracing strategies for COVID-19: a modelling study. Lancet Public Health.

[17] Panovska-Griffiths J (2020). Determining the optimal strategy for reopening schools, work and society in the UK: balancing earlier opening and the impact of test and trace strategies with the risk of occurrence of a secondary COVID-19 pandemic wave. Lancet Child Adolesc Health.

[18] Hale, T. *et al.* Oxford COVID-19 Government Response Tracker*, Blavatnik School of Government.* (2020)

[19] Our World in Data. *Corononavirus Pandemic (COVID-19)* https://ourworldindata.org/coronavirus (2020).

[20] World Deveopment Indicators. *Databank* https://databank.worldbank.org/source/world-development-indicators (2020).

[21] Elgin C, Basbug G, Yalaman A (2020). Economic Policy Responses to a Pandemic: Developing the COVID-19 Economic Stimulus Index. Covid Econ..

[22] Millett GA (2020). Assessing differential impacts of COVID-19 on Black communities. Ann. Epidemiol..

[23] Chaudhry, R. et al. A country level analysis measuring the impact of government actions, country preparedness and socioeconomic factors on COVID-19 mortality and related health outcomes. *EClinicalMedicine*, 100464. (2020). 10.1016/j.eclinm.2020.100464 PMC7372278 32838237

[24] Spychalski P, Błażyńska-Spychalska A, Kobiela J (2020). Estimating case fatality rates of COVID-19. Lancet Infect Dis..

[25] Wang W, Tang J, Wei F (2020). Updated understanding of the outbreak of 2019 novel coronavirus (2019-nCoV) in Wuhan China. J. Med. Virol..

[26] Bhat V (2005). Institutional arrangements and efficiency of health care delivery systems. Eur J Health Econ.

