# Supplementary material for: Cross-country evidence on the association between contact tracing and COVID-19 case fatality rates
Source: Sci Rep. 2021 Jan 25;11:2145. doi: 10.1038/s41598-020-78760-x (PMC7835347; doi:10.1038/s41598-020-78760-x)
Supplement: Supplementary file 1 — Supplementary Information 1. [file 41598_2020_78760_MOESM1_ESM.docx]

**Cross-country evidence for the importance of contact tracing in reducing COVID-19 case fatality rates**

**ABDULLAH YALAMAN**

*Eskisehir Osmangazi University*

Department of Business Administration

Meselik Campus

Eskisehir, 26040, Turkey

[abdullah.yalaman@gmail.com](mailto:abdullah.yalaman@gmail.com)

**GOKCE BASBUG**

*Sungkyunkwan University*

SKK Graduate School of Business

25-2, Sungkyunkwan-ro, Jongro-gu,

Seoul, 03063, South Korea

**CEYHUN ELGIN**

*Bogazici University*

Department of Economics

Natuk Birkan Building

Bebek/Istanbul, TR-34342, Turkey

**ALISON P. GALVANI**

*Yale University*

Center for Infectious Disease Modeling and Analysis

Yale School of Public Health,

New Haven, CT, 06520, USA

**Appendix**

| **Variable** | **Definition** | **Source** |
| --- | --- | --- |
| Diagnostic tests | The number of diagnostic tests conducted per million people | Our World in Data  <https://ourworldindata.org/> |
| Contact tracing | Government policy on contact tracing after a positive diagnosis (0=no contact tracing, 1=limited contact tracing; not done for all cases, 2=comprehensive contact tracing; done for all identified cases) | *Oxford COVID-19 Government Response Tracker* [https://www·bsg·ox·ac·uk/research/research-projects/oxford-covid-19-government-response-tracker](https://www.bsg.ox.ac.uk/research/research-projects/oxford-covid-19-government-response-tracker) |
| The number of hospital beds | The number of hospital beds per 1000 people | World Development Indicators  https://databank.worldbank.org/source/world-development-indicators |
| The number of physicians | The number of physicians per 1000 people | World Development Indicators  https://databank.worldbank.org/source/world-development-indicators |
| Population | Population in 2020 | Our World in Data  <https://ourworldindata.org/> |
| Age over 70 | The percentage of people over 70 years old | Our World in Data  <https://ourworldindata.org/> |
| Diabetes prevalence | The percentage of people with diabetes in the country | Our World in Data  <https://ourworldindata.org/> |
| Smokers | The percentage of smokers in the country | Our World in Data  <https://ourworldindata.org/> |
| Stringency Score | An aggregate score of the intensity of containment measures implemented by country | *Oxford COVID-19 Government Response Tracker* [https://www·bsg·ox·ac·uk/research/research-projects/oxford-covid-19-government-response-tracker](https://www.bsg.ox.ac.uk/research/research-projects/oxford-covid-19-government-response-tracker) |
| Real GDP per capita | Gross domestic product per capita | Our World in Data  <https://ourworldindata.org/> |
| Fiscal stimulus | Fiscal stimulus introduced by governments during pandemic as a percentage of GDP | COVID-19 Economic Stimulus Index  http://web.boun.edu.tr/elgin/COVID.htm |
